# Genetic variation in the serotonin transporter gene (5-HTTLPR, rs25531) influences the analgesic response to the short acting opioid Remifentanil in humans

**DOI:** 10.1186/1744-8069-5-37

**Published:** 2009-07-01

**Authors:** Eva Kosek, Karin B Jensen, Tina B Lonsdorf, Martin Schalling, Martin Ingvar

**Affiliations:** 1 Osher Center For Integrative Medicine, Stockholm Brain Institute, Department of Clinical Neuroscience, Karolinska Institutet, Stockholm, Sweden; 2 Centre for Molecular Medicine, Karolinska Institutet, Stockholm, Sweden

## Abstract

**Background:**

There is evidence from animal studies that serotonin (5-HT) can influence the antinociceptive effects of opioids at the spinal cord level. Therefore, there could be an influence of genetic polymorphisms in the serotonin system on individual variability in response to opioid treatment of pain. The serotonin transporter (5-HTT) is a key regulator of serotonin metabolism and availability and its gene harbors several known polymorphisms that are known to affect 5-HTT expression (e.g. 5-HTTLPR, rs25531). The aim of this study was to investigate if the triallelic 5-HTTLPR influences pain sensitivity or the analgesic effect of opioids in humans. 43 healthy volunteers (12 men, 31 women, mean age 26 years) underwent heat pain stimulations before and after intravenous injection of Remifentanil; a rapid and potent opioid drug acting on μ-type receptors. Subjects rated their perceived pain on a visual analogue scale (VAS). All participants were genotyped for the 5-HTTLPR and the rs25531 polymorphism. We recruited by advertising, with no history of drug abuse, chronic pain or psychiatric disorders.

**Results:**

At baseline, there was no difference in pain ratings for the different triallelic 5-HTTLPR genotype groups. However, the opiod drug had a differential analgesic effect depending on the triallelic 5-HTTLPR genotype. Remifentanil had a significantly better analgesic effect in individuals with a genotype coding for low 5-HTT expression (S_A_/S_A _and S_A_/L_G_) as compared to those with high expression(L_A_/L_A_), p < 0.02. The analgesic effect for the three different genotype groups was linear to degree of 5-HTT expression.

**Conclusion:**

This is the first report showing an influence of the triallelic 5-HTTLPR on pain sensitivity or the analgesic effect of opioids in humans. Previously the 5-HTTLPR s-allele has been associated with higher risk of developing chronic pain conditions but in this study we show that the genotype coding for low 5-HTT expression is associated with a better analgesic effect of an opioid. The s-allele has been associated with downregulation of 5-HT1 receptors and we suggest that individuals with a desensitization of 5-HT1 receptors have an increased analgesic response to opioids during acute pain stimuli, but may still be at increased risk of developing chronic pain conditions.

## Introduction

Modern pain research has highlighted the importance of central nervous system mechanisms for the regulation of acute and chronic pain. The large individual differences in pain sensitivity, response to analgesic drugs and risk of developing chronic pain is partially explained by genetic factors with impact on the endogenous pain modulation that takes place within the central nervous system [1,2]. The activity of the descending pain inhibitory pathways is largely dependent on dopamine/noradrenalin as well as endogenous opioids and previous studies have shown that polymorphisms in genes coding for μ-opioid receptors and the catecholamine-degrading enzyme catechol-*O*-methyltransferase (COMT) influence pain responses [3,4]. However, in addition to well known opioidergic pain regulatory mechanism with a key role of the rostral ventromedial medulla (RVM), there is a separate serotonergic channel from RVM that controls pain transmission in the dorsal horn of the spinal cord in a state-dependent manner [5]. Furthermore, there is evidence from animal studies that serotonin (5-HT) can influence the antinociceptive effects of opioids at the spinal cord level [6,7]. This means that there may be a potential influence of functional genetic polymorphisms of the serotonergic system on individual variability in response to opioid treatment of pain.

The serotonin transporter (5-HTT) is a key regulator of serotonin metabolism. Localized in pre-synaptic neuronal membranes it transports serotonin from the synapses back into the presynaptic neuron thus terminating its synaptic actions [8]. The human 5-HTT is encoded by one single gene, *SLC6A4*, which contains several known polymorphisms in its promoter region that affect the transcriptional efficacy of the *5-HTT *gene e.g. 5-HTTLPR (5-HTT linked polymorphic region) [9] and rs25531 [10]. The 5-HTTLPR consists of a 43-bp insertion/deletion yielding a short allele (s) and a long allele (l). The s-allele reduces the transcriptional efficiency of the 5-HTT gene promoter, resulting in decreased 5-HTT expression and availability [9]. The s-allele has been reported to affect the cerebral response to emotional stimuli [10] and to be associated with higher neuroticism [11]. In the clinical context, the s-allele has been associated with anxiety disorders [12], substance abuse [13] as well as chronic pain syndromes such as fibromyalgia [14], irritable bowel syndrome [15] and tension type headaches [16]. A single-nucleotide polymorphism (rs25531) located in close proximity of the 5-HTTLPR leading to an A to G substitution has been shown to further modulate the effect of the 5-HTTLPR on transcriptional efficacy. The minor G-allele is almost always in phase with the 5-HTTLPR long allele and reduces the transcriptional efficacy to the level associated with the 5-HTTLPR s-allele. Both polymorphisms are commonly studied jointly and referred to as triallelic 5-HTTLPR differentiating between individuals with a high (L_A_/L_A_), intermediate (L_A_/L_G_, S_A_/L_G_) or low (S_A_/S_A_, L_G_/S_A_) transcriptional efficacy. Despite the fact that the SNP rs25531 is separate from the LPR and thus technically we genotype two separate polymorphisms in strong LD, we have chosen to use the established term "triallelic 5-HTTLPR" to indicate that we are performing the same analysis as in previous publications using this term [17].

Drugs inhibiting the 5-HTT and the norepinephrine transporter (NET) (i.e. serotonin and norepinephrine re-uptake inhibitors (SNRIs)) have antidepressive and anxiolytic effects, but also have antinociceptive effects [18-22]. Furthermore, SNRIs and, to a more limited extent, also selective serotonin re-uptake inhibitors (SSRIs) have analgesic effects in patients with neuropathic pain [23] and fibromyalgia [24,25] two conditions associated with changes in the function of endogenous pain modulation and reports of insufficient analgesic response to opioids [26,27]. Given the efficacy of serotonergic drugs in the treatment of pain, genetic polymorphisms in the serotonergic system may contribute to individual variability in antinociceptive effects after opoid administration.

To our knowledge, no previous study has addressed the possible influence of the triallelic 5-HTTLPR on pain sensitivity or the analgesic effect of opioids in humans. The aim of this pilot study was to investigate if; 1) heat pain sensitivity, and 2) the relative analgesic effect of a short acting opioid, differed between healthy subjects dependent on the triallelic 5-HTTLPR genotype.

## Methods

Participants were 43 healthy subjects (12 men and 31 women, mean age 26 years, SD 5), recruited by advertising. To meet the inclusion criteria subjects had to be over 18 years old, right-handed, take no medications (female subjects were allowed to take contraceptive pills), have no history of drug abuse, chronic pain or psychiatric disorders. The psychiatric history was assessed by subjects' self-report and not by formal interview. Anxiety dimensions were addressed by the SpielbergerTrait Anxiety Inventory (STAI-T) [28]. Subjects were recruited from a variety of institutions in order to represent all sorts of educational and professional backgrounds. All subjects were Caucasian. The study was conducted according to the principles expressed in the Declaration of Helsinki. The study was approved by the Institutional Review Board of Karolinska Institutet (Reference number 2005/950-31/1) and all patients provided written informed consent for the collection of samples and subsequent analysis.

Subjects went through an experiment where pain was induced to the dorsum of the left hand using a 3 × 3 cm heat probe (Medoc TSA, Medoc Israel). In total two blocks of 30 seconds of tonic heat (48°Celsius) were administered at two different positions on the subjects' hand with a ten minute interval. The order of the two positions on the hand were counter-balanced. After each block subjects were asked to rate the pain on a visual analogue scale (VAS) ranging from 0 mm (no pain) to 100 mm (worst imaginable pain). An intravenous injection of a short acting and potent opioid acting on μ-receptors i.e., Remifentanil (4-(Methoxycarbonyl)-4-[(1-oxopropyl) phenylamino]-1 – piperidine propa-noic acid methyl ester) was given 15 seconds before the second heat stimulation block. The administration of the opioid was overt and before the injection the subject was informed that this would be a fast acting opioid drug. The subject was also informed that the effect of the drug would last approximately for the duration of the stimulation and then disappear. Remifentanil has a rapid onset of action and a very short duration. The time for a 50% reduction in the effect site concentration is about 3.65 min and the terminal elimination half-life 10.2 min at a dose of 2 μg/kg. The minimal effective dose of Remifentanil in healthy controls in our laboratory setting was determined in a previous pilot study and amounted to 0.08 μg/kg body weight, which was used in this study. During the experiment the subject and experimentator were blinded as to the genetic category of the subject.

### Genotyping

Samples of 20 ml whole blood were taken venously and stored at -80°C until DNA extraction, which was performed robotized by the local Biobank (KI Biobank, Karolinska Institutet, Stockholm) using standard methods. (Autopure LS system [Gentra Systems, Minneapolis, MN]). DNA yield was measured with UV at 260 nm and with the 260/280 ratio as a quality check. Genotyping for 5-HTTLPR was performed as described in detail earlier [29]. For the triallelic 5-HTTLPR a different protocol was used and PCR reactions were performed in a final volume of 20 μl containing 50 ng of genomic template, 0.2 mM each deoxynucleoside triphosphate (dNTP), 0.4 mM each primer, 0.05 U/μl Quiagen HotStar^®^Polymerase, 1 M Q-Solution and 1× Buffer. The primer (Thermo Scientific, Ulm, Germany) sequences were Forward: 5'-GGCGTTGCCGCTCTGAATGC-3' and Reverse. 5'-GAGGGACTGAGCTGGACAACCAC-3'. Samples were amplified on a Biorad Tetrade (BIORAD, Hercules, CA, USA) with an initial denaturation step for 10 min at 94°C followed by 32 cycles consisting of denaturation for 30 s at 95°C, annealing for 30 s at 57°C and elongation for 30 s at 72°C and one final elongation step for 5 min at 72°C. This yields a "short" 486 bp and a "long" 529 bp fragment. 8 μl of the PCR product separated for 2 h at 180 V on a 2.5% agarose gel containing GelRed^® ^and visualized using UV light. 10 μl of the remaining PCR product were digested for 12 h at 37°C with 0.1 μl MSP1 (New England Biolabs, Ipswich, MA, USA) and 1 μl buffer per sample. MSP1 recognizes and cuts a 5'-C/CGG-3' sequence resulting in the following fragments: 340 bp, 127 bp and 62 bp for the L_A _allele, 297 bp, 127 bp and 62 bp for the S_A _allele, 174, 166, 127 and 62 bp for the L_G _allele and 166, 131, 127 and 62 bp for the S_A _allele. Fragments were run for 2 h at 180 V on 4% Agarose gels containing GelRed^® ^and visualized via UV light. All biallelic 5-HTTLPR Genotypes were thus determined using two different protocols that yielded identical results.

### Statistics

We wanted to investigate the influence of the three different triallelic 5-HTTLPR genotypes coding for low, medium or high 5-HTT expression on the relative analgesic effect of Remifentanil. Therefore, the analysis of pain ratings at baseline was based on raw scores, but when investigating the relative effect of Remifentanil the pain ratings were normalized in order to control for inter-individual differences. The normalized VAS ratings were calculated by dividing the individual VAS rating following Remifentanil by the individual VAS rating at baseline. The differences between the three different triallelic 5-HTTLPR genotypes in VAS ratings of heat pain at baseline and in normalized VAS ratings following Remifentanil were analyzed by Kruskal-Wallis Tests. When significant differences were found, the Mann-Whitney U test was used to perform individual group comparisons. Intragroup differences in VAS ratings before and after administration of Remifentanil were analyzed by Wilcoxon's signed rank test. Sex differences in heat pain sensitivity at baseline and in normalized VAS ratings following Remifentanil were analyzed by Mann Whitney U-test. A p-value of < .05 was considered statistically significant (two-tailed tests).

## Results

Genotyping yielded the following distribution for triallelic 5-HTTLPR: 12 individuals homozygous for the long allele (all L_A_/L_A_), 13 heterozygotes on a rs25531 A-allele background (S_A_/L_A_), four heterozygotes with an L_G _allele (S_A_, L_G_) and 10 individuals homozygous for the short allele, all of which on a A-allele background (S_A_/S_A_) (see Table 1). When grouping individuals based on their putative transcriptional efficacy, we compared 12 individuals with a genotype coding for a high 5-HTT expression (L_A_/L_A_) with 17 individuals with intermediate expression (L_A_/S_A_) and 14 low expressing individuals (S_A_/S_A _and S_A_/L_G_). Genotype distributions for 5-HTTLPR did not differ significantly from those predicted by the Hardy-Weinberg equilibrium (HWE), chi^2 ^= 0.02, p = 0.89. The distribution of the rs25531 polymorphism did not differ from HWE either; exact test, p = 1.0. The number of male/female subjects for each of the genotype groups is shown in table 1.

**Table 1:** Distribution of the triallelic 5-HTTLPR genotypes for men and women in the present sample (n = 43).

	**High expressing** **(L_A_/L_A_)**	**Intermediate expressing** **(L_A_/S_A_)**	**Low expressing** **(S_A_/S_A _and L_G_/S_A_)**
**Male**	4	3	5
**Female**	8	14	9

**Total**	12	17	14

Participants in the triallelic 5-HTTLPR genotype groups did not differ significantly from each other in their trait anxiety as assessed by the Spielberger Trait Anxiety inventory [28], F(2,36) = 2.28, p = 0.12.

**Figure 1:**
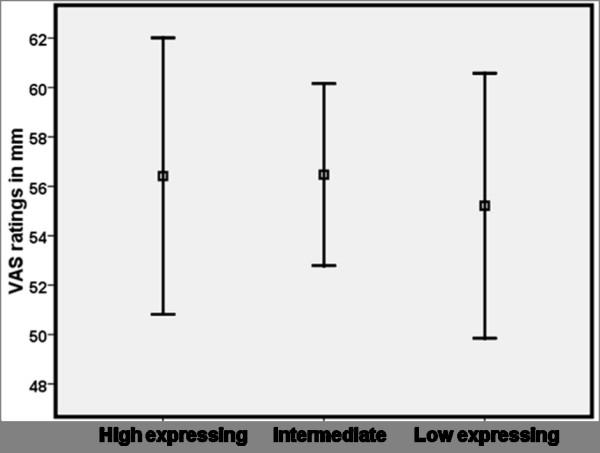
**Average ratings of heat pain intensity (mm VAS) ± SEM during 30 seconds of tonic contact heat stimuli corresponding to 48°Celsius in 43 healthy individuals grouped according to the triallelic 5-HTTLPR genotypes, putative high (n = 12) intermediate (n = 17) and low 5-HTT expression (n = 14)**. There were no statistically significant differences between the genotype groups in heat pain sensitivity at baseline.

At baseline, there were no statistically significant differences between the three triallelic 5-HTTLPR genotypes in heat pain sensitivity (see Figure 1). In order to validate the experimental manipulation, we confirmed that the dose of Remifentanil used had an analgesic effect in our study population, W = -2.92, p < 0.005.

**Figure 2:**
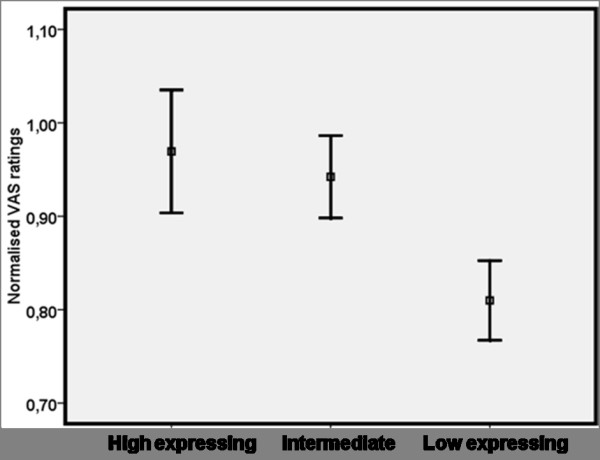
**Normalized heat pain ratings following administration of Remifentanil (0.08 μg/kg body weight) intravenously (average ± SEM) during 30 seconds of tonic contact heat stimuli corresponding to 48°Celsius in 43 healthy individuals grouped according to the triallelic 5-HTTLPR genotype, putative high (n = 12) intermediate (n = 17) and low 5-HTT expression (n = 14)**. VAS ratings were normalized by dividing the individual VAS heat pain rating following Remifentanil with the individual VAS rating at baseline. Remifentanil had a significantly better analgesic effect in subjects with low 5-HTT expression (S_A_/S_A _and S_A_/L_G_) compared to subjects homozygous for the 5-HTTLPR L_A _allele (p < 0.02). Subjects with low 5-HTT expression rated the heat stimuli as significantly less painful following Remifentanil compared to baseline (p < 0.001). No statistically significant analgesic effect of Remifentanil was seen in the other two genotype groups.

For normalized VAS ratings, the triallelic 5-HTTLPR genotype had a significant main effect on the analgesic effect of the opiod drug, chi^2 ^= 6.28, df = 2, p < 0.05 (See figure 2). Remifentanil had a significantly better analgesic effect in subjects with a genotype coding for low 5-HTT expression (S_A_/S_A _and L_G_/S_A_) compared to subjects with high expression (L_A_/L_A_), chi^2 ^= 6.01, df = 1, p < 0.02. The heterozygous subjects (L_A_/S_A_) had an intermediate response to Remifentanil and did not differ significantly in analgesic response from either low, chi2 = 2.73, df = 1, p < 0.10 or high expressing individuals, chi2 = 0.91, df = 1, p < 0.35.

The analgesic effect for each of the three genotype groups was further explored in three separate analyses. Only subjects homozygous with a genotype coding for low 5-HTT expression (S_A_/S_A _and L_G_/S_A_) had a significant analgesic effect of Remifentanil compared to baseline (p < 0.001), while no statistically significant analgesic effect of Remifentanil was seen in the other two genotype groups.

Heat pain ratings at baseline were higher in female subjects (average: 60 mm, range: 20 – 85 mm) compared to male subjects (average: 46 mm, range 24 – 70 mm), chi^2 ^= 6.28, df = 1, p < 0.02, but there were no statistically significant sex differences in the normalized VAS ratings following administration of Remifentanil (female average 0.92, range 0.56 – 1.33; male 0.86, range 0.38 – 1.26), chi^2 ^= 0.19, df = 1, p = 0.66.

## Discussion

We found that the triallelic 5-HTTLPR is associated with individual differences in analgesic response to an opioid drug in healthy subjects. Individuals with a genotype coding for low 5-HTT expression (S_A_/S_A _or S_A_/L_G_) responded with more pain relief to Remifentanil than individuals with a genotype coding for high (L_A_/L_A_) 5-HTT expression. Interestingly we did not observe any difference in pain sensitivity between the genotype groups at baseline, which is in accordance with studies showing no difference in the response to heat pain stimuli in 5-HTTLPR knock-out and wild type mice [30,31]. To our knowledge, this is the first report of an influence of the 5-HTTLPR polymorphisms on pain sensitivity in humans.

Serotonin has complex actions on nociception, with both pro- and antinociceptive effects and differential effects depending on the receptor type that is activated [32]. For example, 5-HTTLPR knock-out mice have increased extracellular levels of serotonin but the overall tissue content is reduced [33,34]. Furthermore, complex compensatory mechanisms with upregulation of certain 5-HT receptors (i.e., 5-HT3) and down-regulation and/or decreased function of others (i.e. 5-HT2a and 5-HT1a, b) has been reported in 5-HTTLPR knock-out mice which most likely explains the lack of differences in pain sensitivity at baseline [31]. Our data indicate that this may also be true for the 5-HTTLPR polymorphisms in humans, although the sensitivity to other modalities than heat pain remain to be investigated as well as sensitivity to longer durations of pain stimuli. In accordance with previous studies we found higher pain sensitivity in females compared to male subjects at baseline [35,36]. However, the relative analgesic effect of Remifentanil did not differ between male and female subjects.

The low dose of Remifentanil used in our study had a significant analgesic effect that was further qualified by the triallelic 5-HTTLPR genotype. Subjects with a genotype coding for low 5-HTT expression (S_A_/S_A _and S_A_/L_G_) rated the heat stimuli as significantly less painful following Remifentanil compared to individuals with high expression (L_A_/L_A_) and those with an intermediate expression showed an intermediate response. These findings may seem contra-intuitive since individuals with the 5-HTTLPR s-allele are overrepresented in various chronic pain conditions [1,8] but our results are in accordance with previous reports from animal studies investigating the mechanisms involved in pain processing and analgesia. There are many types of serotonin receptors but the 5-HT1a receptor plays a significant role in mediating pain regulatory effects. Down-regulation of the function of serotonin auto-receptors 5-HT1a and b have been documented in 5-HTTLPR knock-out mice [34,37] and desensitization of the 5-HT 1a and b receptors is believed to be of importance for the effects of antidepressive drugs [38] inhibiting 5-HT reuptake. Individuals carrying an s-allele have a reduced 5-HTT activity and therefore a compensatory decrease in 5-HT1 activity would be expected [39]. There is evidence from animal studies that 5-HT1a agonists inhibit opioid release in the dorsal horn of the spinal cord [7] and reduce the analgesic effects of morphine [40]. Therefore increased analgesic effect of opioids, as seen in our study in individuals with a triallelic 5-HTTLPR genotype coding for low 5-HTT expression, would be the expected result of a desensitization of 5-HT1a receptors. However, whereas 5-HT1a agonists have a hyperalgesic effect during acute pain stimulation, they are known to have analgesic effects during tonic painful stimulation [41] Accordingly, 5-HT1a agonists have been shown to have analgesic effects in animal models of nociceptive [40] as well as neuropathic [42,43] pain, and the analgesic effect increases with the duration of pain [41]. This means, hypothetically, that individuals with a desensitization of 5-HT1 receptors would have an increased analgesic response to morphine and endogenous opioids during acute pain stimuli, but may still be at increased risk of developing chronic pain conditions. Our suggestion is supported by a brain imaging study from 2007 [44] showing that subjects with high availability of 5-HT1a receptors exhibit low pain intensity accompanied by a high capacity for central suppression of pain during a tonic pain provocation, i.e., experimental cold pressor pain. Considering these results, it would be of interest for future studies to investigate a possible impact of genetic variation in the gene coding for the 5-HT1a receptor (e.g. 5HT1A -109C/G) on opoid induced analgesia.

On the first glance, our results seem to contradict the results of Park et al. [45] who observed in a sample of Korean patients suffering from chonic tension-type headache that patients with the biallelic 5-HTTLPR s/s genotype (N = 76) use significantly more analgetic drugs than patients carrying at least one 5-HTTLPR l-allele (N = 22). The results are interpreted as defective pain control in patients with the s/s genotype. However our study may offer an alternative explanation for their results. We propose that carriers of the 5-HTTLPR l-allele use less analgesic drugs simply because they may not profit from their analgesic effect to the same amount as patients with the s/s genotype do. Furthermore Park et al. argue for an "overuse" of analgesics seen in patients with the s/s genotype but as more than 70% of the sample had this genotype they probably show a normal consumption while carriers of the l-allele show a reduced consumption.

Our study had several limitations. The sample size was small and the results from this study should therefore be considered preliminary until there is validation from future studies. Larger samples are needed to specifically address the impact of the variations of the serotonin transporter gene on opioid analgesia in relation to gender and mood. Furthermore, the design of our study did not permit a clear distinction between the analgesic effects of endogenous opioids (due to expectation) from the effect of the exogenous opiod, since both form part of the same opioidergic physiological network [46].

In conclusion, we found that healthy subjects with the triallelic 5-HTTLPR genotype coding for low 5-HTT expression (S_A_/S_A _and S_A_/L_G_) had a better analgesic effect of a short acting opioid drug compared to those homozygous for the 5-HTTLPR L_A _allele. The baseline sensitivity to heat pain was not affected by the triallelic 5-HTTLPR polymorphism. Results from this exploratory study show that the analgesic effect of a short acting opioid is influenced by the triallelic 5-HTTLPR polymorphism in healthy humans and the potential clinical implications of this finding need to be explored.

## Competing interests

The authors declare that they have no competing interests.

## Authors' contributions

EK participated in the design of the study, performed the statistical analysis and drafted the manuscript. KBJ participated in the design of the study, performed the behavioral experiments and helped to draft the manuscript. TBL carried out the genotyping, helped with statistical analyses and to draft the manuscript. MS contributed with genetic expertise and design of polymorphism assays used in paper. MI conceived the study, contributed with coordination of the experiments and helped to draft the manuscript. All authors read and approved the final manuscript.
